# Calves fed more milk prioritise play over feeding in a hole-board test

**DOI:** 10.1038/s41598-026-47403-y

**Published:** 2026-04-13

**Authors:** Jillian Hendricks, Thomas Leroy, Michael T. Mendl, Benjamin Lecorps

**Affiliations:** Bristol Veterinary School, University of Bristol, Bristol, BS40 5DU UK

**Keywords:** animal welfare, animal wellbeing, dairy cattle, food motivation, positive animal welfare, Ecology, Ecology, Neuroscience, Zoology

## Abstract

Dairy calves are typically feed-restricted. Depending on its severity, hunger can improve or impair animals’ cognitive abilities, but the effect of chronic feed restriction on calf cognition remains underexplored. We used a spatial foraging task (hole-board test) to assess whether chronic feed restriction impairs cognitive performance in dairy calves provided 6 L/day of milk (restricted) compared to calves provided up to 12 L/day (enhanced). Calves had to recall 4 milk reward locations among 15 possibilities; locations remained constant for 14 trials (initial learning), and were then changed and kept constant for 6 trials (re-learning). We measured calves’ ability to remember locations already visited within trials (working and general working memory) and recall reward locations between trials (reference memory). We also measured play behaviour to explore trade-offs between foraging and playing in the testing arena (larger than calves’ home-pen). Restricted calves had better working and reference memory, shorter latencies to reach the first bucket, and completed trials faster, indicating higher food motivation. Enhanced calves were less motivated to engage in the test but played more than restricted ones. This highlights how hunger can shift priorities to foraging. Future research should explore trade-offs between important behaviours like play and feeding to understand the value calves place on different activities.

**Supplementary Information:**

The online version contains supplementary material available at 10.1038/s41598-026-47403-y.

## Introduction

Feed restrictions are common in dairy calf management systems. For example, in the UK pre-weaned dairy calves are typically fed 4–6 L/day^1^ but by 3 weeks of age calves fed *ad libitum* consume an average of 10 L/day^2^ and by 6 weeks old intake reaches 16 L/day^3^. Calves are also frequently weaned earlier and more abruptly compared to the natural process, despite evidence that calves fed higher volumes of milk display greater body weight gain during pre-weaning^4^ and improved feed efficiency^5^. On the other hand, greater milk allowances have mixed effects on calf health; a review by Khan et al.^5^ reported reduced incidence of disease, while a review by Welk et al.^4^ found little effect. Feed-restricted calves display behavioural indicators of hunger, including an increase in unrewarded visits to the feeder^4^, time spent sucking on the teat (nutritive and non-nutritive), and competitive behaviours at the feeder^6^. Thus, research suggests that feed restriction in pre-weaned calves can have a negative impact on various physiological and behavioural indicators of welfare.

Despite evidence of reduced welfare due to feed restriction, physiological and behavioural indicators alone provide limited information about affective states associated with hunger. In humans, hunger is recognised as a state that gives rise to negative, high arousal emotions (triggered by the release of hormones such as ghrelin and cortisol) and can suppress positive emotions^7^, sometimes being described using the colloquial expression *hangry*^8^. Although non-human animals are also expected to experience negative affect when hungry^9^, the nature of these states are poorly understood. Given that an animal’s affective states are increasingly viewed as key determinants of their welfare^10^, understanding calves’ subjective experience of hunger is necessary to gain the full picture of how feed restriction impacts their wellbeing.

Cognitive functioning can provide useful information about affective states; both human and animal literature have shown biases in cognitive processes (e.g., judgement, memory, and attention) that vary according to emotional state^11–13^. One example that has been studied extensively is judgement bias, where animals presumed to be in a negative affective state may demonstrate ‘pessimistic-like’ responses to ambiguous stimuli^14–16^. Other types of cognitive functioning that appear to be influenced by affective state include aspects of reward learning (e.g.^12,17^. Reward learning can be assessed using spatial foraging tasks such as the hole-board test, in which animals search for food amongst a number of possible locations, which provides information on the animal’s capacity to encode and recall memories about food location. Short-term working memory relates specifically to the ability to avoid re-visiting food locations within trials, and general working memory performance can be quantified in terms of how well they learn to avoid locations (regardless of whether they contain food) that they have already visited within trials. In contrast, long-term reference memory performance is quantified in terms of the animal’s ability to remember where the food locations are from one day to another^18^. In male rats, reference memory in a hole-board task is consistently impaired by chronic social stress, whereas working memory is transiently affected and is more likely to recover quickly^19^. However, impairment of any aspect of memory would have detrimental effects on animal welfare as it may lead to poor learning abilities, disrupted memory retrieval, and subsequent behavioural problems such as aggression towards familiar individuals^20^.

Hunger motivates animals to seek and acquire food, which leads to increased exploration and foraging behaviour^21,22^. In a foraging task, high motivation to find food may lead animals to perform better when hungry compared to when satiated^23,24^. However, whilst hunger can be a motivator in cognitive tasks involving food, severe hunger can act as a stressor and impair cognitive functioning (see Mendl^25^ for the relationship between stress and cognitive performance). Chronic feed restriction in particular may cause cognitive impairments; for example, in broiler chickens it impairs their ability to learn a feed quantity discrimination task^26^, and in mice, chronic restriction of 30–50% of *ad libitum* impairs spatial recognition memory in a Y-maze task^27^. Thus, the relationship between hunger and cognitive performance in spatial foraging tasks can be visualised as an inverted U-shape; animals should exhibit optimal cognitive performance when they are hungry enough to be sufficiently motivated, and lower or higher levels of hunger may lead to impaired performance.

Recent evidence shows that calves who experience acute hunger induced by a sudden reduction in their milk allowance (mimicking what happens at weaning) exhibit impaired working and reference memory^28^. However, less is known about whether chronic milk restriction affects calves’ cognitive performance. The objective of this study was to assess whether standard feed restrictions (6 L/day) impaired cognitive performance in a hole-board test compared to enhanced feeding (up to 12 L/day), by assessing calves’ ability to (1) learn a pattern of milk reward locations (initial learning), and (2) re-learn a different pattern after the locations of the rewards were changed (re-learning). We anticipated that feed restriction would cause calves to experience a higher arousal and more negative emotional state and thus impair cognition, such that calves fed a restricted diet would demonstrate deficits in working memory, general working memory, and reference memory during both the initial learning and the re-learning phases.

Dairy calves are highly motivated to play when brought into a novel arena, especially when play behaviours are restricted in their home-pen due to the limited amount of space provided^29–31^. Given the nature of the hole-board apparatus used in this study, we expected that their engagement in the task may conflict with the motivation to express play behaviours. Feed restriction is known to reduce play in calves^32,33^, so we expected that feed-restricted calves would demonstrate reduced play behaviour compared to enhanced-fed calves.

## Methods

This study was approved by The University of Bristol AWERB Committee (# UIN-22-020). Consistent with the principles of the 3Rs (https://nc3rs.org.uk/), the number of animals used in this study was kept to a minimum with power calculations and efficient experimental designs. The restricted milk allowance used in this study corresponds to standard farm practices, so calves did not experience worse conditions due to being enrolled. This experiment used the ARRIVE guidelines consistent with best practices in sharing information in scientific publication (Supplementary Material File 1).

### Animals and Housing

Calves were housed at the University of Bristol dairy farm following the farm’s standard practices. Calves were fed 4 L of colostrum (Brix value > 22%) within 6 h of birth, and then another 4 L 12 h after the first colostrum feed. Separation from the dam occurred 24 h after birth. Calves were then housed in pairs in hutches (2.5 m x 1.5 m) bedded with straw that was topped up once daily, combined with a Flexyfence (Calf Igloo, Maghera, Northern Ireland, UK) providing 5.8m^2^ of outdoor access. Calves were fed 2.5 L of milk replacer (Sprint Plus 50, Bridgmans Farm Direct, Shepton Mallet, UK; 190 gl^–1^) from buckets with attached teats (Super Red 100 mm, Mole Valley Farmers, South Molton, UK) twice per day (approximately 7:00am and 4:30pm) until the first day of the experiment. Calves were given *ad libitum* access to water, straw, and grain (18% Premium Calf, Tamar Milling, Whitstone, UK).

### Apparatus

**Fig. 1:**
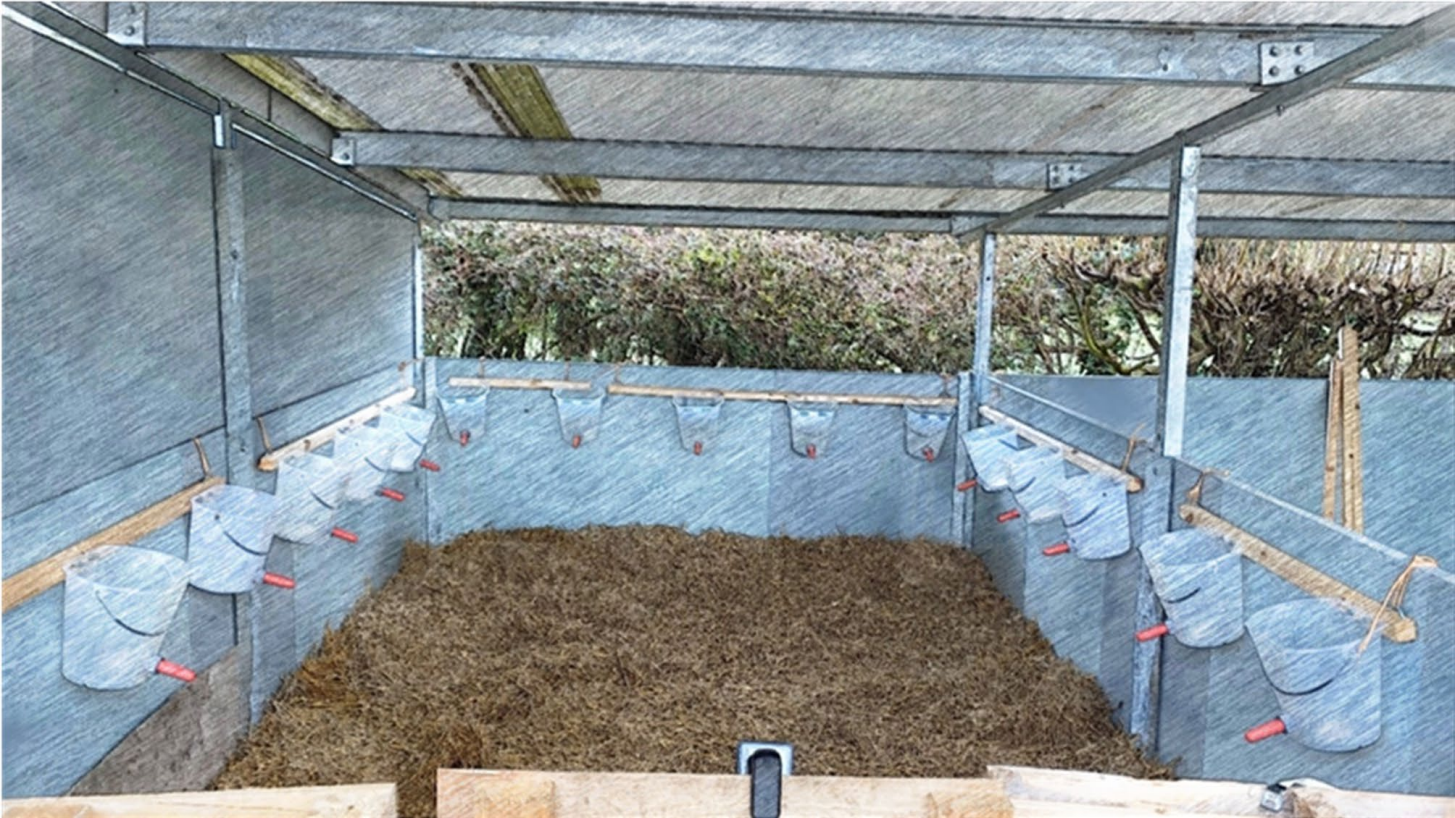
Hole-board apparatus view from the entrance door. The test used 15 buckets (5 on 3 walls), and 4 were baited with milk. Calves were tested individually for a maximum of 5 min. Sketch effect was applied to the image using FotoSketcher v3.98.

The hole-board apparatus was a 5 m x 3 m roofed arena connected to a start box via a latched door (see Fig. 1). Fifteen buckets (5 on 3 sides of the apparatus) were affixed to 3 walls (both sides and rear walls). Baited and unbaited locations consisted of milk-filled (0.5 L) and empty milk buckets, respectively. Milk was visible through the buckets. This was essential to determine whether calves drank the full reward (revisits would then be considered an error) or not (revisits would not be considered an error). Work from Lecorps et al.^34^ determined that calves do not rely on sensory cues (i.e., vision and olfaction) to locate rewarded buckets in a similar test. Each bucket teat was 0.65 m apart and approximately 0.8 m from the ground. The arena was straw-bedded and straw was added or cleared out and replaced as needed. In front of the arena, a 4.1m^2^ fenced start box was used to allow calves to enter the arena freely.

## Experimental procedures

### Treatments and management

We compared the cognitive performance of calves that were fed a restricted amount of milk (6 L/d) to calves on an enhanced milk allowance (up to 12 L/day). 6 L/d was chosen to maintain consistency with common practice in the UK to feed calves 4–6 L/d^1^. Twenty (13 Longhorn, 4 Holstein Friesian, and 3 British Blue) pre-weaned dairy or dairy beef crossbred calves (14 females and 6 bulls) were used in this experiment. Calves were housed in pairs based on age and enrolled in the experiment at 10 (+/- 2) d of age, following the enrolment age protocol of Yoo et al.^35^. Calves were included if they were healthy on the scheduled day of enrolment (i.e., drinking well and no signs of disease). If the calf was sick, enrolment was delayed until the calf was deemed healthy. Treatment was assigned a priori and pseudo-randomly, with one calf in each pair assigned to the enhanced treatment and the other to the restricted treatment. Distribution of breed and sex across treatments was as follows: Enhanced – 6 females and 4 males; 2 Holstein Friesian, 2 British Blue, and 6 Longhorn. Restricted – 8 females and 2 males; 2 Holstein Friesian, 1 British Blue, and 7 Longhorn.

On the day of enrolment (which was also the day that calves underwent their first training session), calves fed the enhanced diet were gradually introduced to their new, increased milk allowance. Quantity was increased by 1 L each time calves finished their meal, and if they did not finish, milk allowance at the next meal was only increased by 0.5 L, until reaching 12 L/day by the end of training. Calves fed a restricted diet remained on their standard milk allowance of 6 L/day. All calves were fed from the same teat buckets used prior to enrolment in the experiment. The calves’ pens were checked for diarrhoea twice per day and if required, one cap of Rehydion gel (Ceva Animal Health Ltd., Wooburn Green, England, UK) was added to every 3 L of milk until diarrhoea resolved. When calves presented with a heightened temperature (> 39.5 °C), and no motivation to drink, testing was stopped until recovery and the calf was given 2 L of water with Prolyte Extra for two subsequent feedings if recommended by the farmers and/or veterinarians. No calves were removed from the experiment, and calves were enrolled in the experiment for 26 (+/- 1.3) days.

### Stage 1: training

Calves were trained twice daily at approximately 8:00am and 4:00pm based on procedures used in Lecorps et al.^34^. In session 1, calves were brought to the arena individually and allowed to drink milk from a single bucket containing their full milk meal. In session 2, 3 baited buckets (0.5 L milk) were presented so that calves learned that there could be several baited bucket locations. In session 3, 6 baited buckets were displayed in the arena (pseudo-randomly located; 2 buckets on each side). To proceed to the final step, calves had to find and drink from at least 5 of the 6 baited buckets. This allowed calves to become familiar with switching to a new bucket once one was emptied. In session 4, calves proceeded to the last training step where only 3 of 6 buckets were baited and the remaining 3 buckets were empty. Calves passed this training stage if they drank milk from the 3 baited buckets and visited at least 2 empty buckets (visiting 5/6 buckets in total). Visiting empty buckets ensured that calves were familiar with unbaited buckets and would continue visiting buckets to find the baited locations during testing. Once the calves achieved this criterion, they proceeded to the testing phase.

### Stage 2: testing

Calves were tested for 20 trials to find 4 baited bucket locations among the 15 available buckets. Testing occurred once daily (excluding Sundays) immediately prior to their morning feeding (approximately 8:00am). Baited locations remained constant during this phase and were matched pairwise (within each pair of calves). For each pair, baited bucket location was pseudorandomised (using a random number generator for locations 1–15 and for allocation of one wall to hold two baited buckets). On trial 15, the locations of the baited buckets were changed and thereafter kept constant for 6 trials to assess calves’ ability to re-learn the new locations of baited buckets. The new baited locations were randomised using the same process but baited locations used in the first phase were excluded from allocation in the second phase. The order of testing proceeded from the youngest pair of calves to the oldest to minimise disease transmission. Within pairs, order of testing was balanced; for 5 pairs, enhanced calves were tested first, and for 5 pairs restricted calves were tested first. We tested 1–3 pairs per day depending on the number of calves enrolled at a given time, and bucket teats were cleaned with water in between each trial. On the first day of testing calves were 13 (+/- 2) days old.

Calves were guided from their home pen to the start box by offering 1 L of milk in a bottle as an incentive. This process ensured that they were focused on getting milk before commencing the test. Once calves were in the start box, the bottle was removed, the door was opened, and calves were allowed to enter the arena freely. If calves did not enter the arena on their own, they were gently pushed into it. A ‘visit’ to the bucket was defined as when the calf touched the teat of any bucket with their nose or mouth. Visits were scored as ‘rewarded’ if the calf drank milk from the bucket, and buckets were recorded as empty when milk was no longer visible in the bucket, and when the sound of sucking indicated that the calf began to suck in air rather than milk. Trials were ended if (1) the calf found and drank from (but not necessarily emptied) all 4 baited buckets; or (2) 5 min elapsed. Trials were recorded using a GoPro camera (Hero 9).

Calves were fed twice daily at approximately 8:15am and 4:30pm. During training (mornings and evenings) and testing (mornings only) feedings took place immediately after calves completed the session and were returned to their home-pen. At the time of morning training and testing, calves had been feed-deprived for 16 h consistent with farm practice and with previous work where calves were feed-deprived for 10–16 h before hole-board testing^28,34^. After training and testing, the milk drunk during the session was subtracted from calves’ treatment-based milk allowance (either 3–6 L per feeding) and the remaining quantity was given in the home-pen. During the testing phase calves’ evening meal took place at 4:30pm and they were fed 3–6 L according to their treatment. Calves in the restricted treatment drank an average of 5.9 L (+/- 0.05) per day, and calves in the enhanced treatment drank an average of 9.5 L (+/- 0.68) per day.

### Data collection and analysis

We recorded calves’ sequence of bucket visits, number of baited buckets found, latency to the first bucket (time from the calf’s whole body entering the arena to the time of the first bucket visit), trial duration (the time taken to find all 4 baited buckets or the maximum trial duration (5 min) if the calf did not find all the baited buckets), and vocalisations during the trials. This data was recorded live so observers were not blind to calves’ treatments. Observers were trained on live scoring during pilot testing of 3 calves who were not included in the main sample.

Measures of cognitive performance were assessed based on definitions from van der Staay et al.^18^. From the sequence of buckets visited, we calculated *working memory* based on visits and revisits to baited buckets (the number of rewarded visits divided by the total number of visits to the baited buckets) and *general working memory* based on visits and revisits to all buckets (the number of different buckets visited divided by the total number of visits), to determine how well calves remembered which buckets they have already visited within a trial, an indicator of short-term memory. We also calculated *reference memory* (the number of baited buckets visited divided by the total number of visits), to determine how well calves recalled the location of baited buckets across trials, an indicator of long-term memory. We adjusted working and reference memory to reflect the number of baited buckets that calves found to account for calves that were less motivated to find rewards; the memory score was multiplied by the proportion of baited buckets found (out of 4) and these new scores were used for subsequent analysis.

Play behaviours were measured using an ethogram adapted from Jensen and Kyhn^29^, which included time spent running, number of jumps, bucks, and head shakes (see Supplementary Material File 2 for ethogram). Some calves often ran between buckets to move to the next location. Therefore, play running was distinguished from running to the next bucket when at least 3 s passed before the next visit; if the calf visited a bucket within 3 s of running, this was not counted as play. Due to low frequency of jumps, bucks, and head shakes, these behaviours were summed into a single measure labelled ‘point play behaviour’. We transformed raw play running into ‘time spent running per minute’ to account for variation in calves’ trial duration and our criteria to end trials when the calf found all baited buckets (thus giving them less time to play). Point play was not transformed due to GLMM analysis (see ‘statistical analysis’ section). The full dataset can be found in Supplementary File 3.

Video recordings of testing trials were analysed by two blinded and trained observers using Boris^36^. Inter-observer reliability for play behaviour was determined using a random subset of 16 videos to calculate the intraclass correlation coefficient in R (ICC: two-way random effects, absolute agreement, two raters, psych package v2.4.6.26; see Supplementary Files 4 and 5). There was a range of behaviours performed across the videos, from no behaviours performed to all behaviours performed multiple times. Reliability for duration of play running and number of jumps was excellent (ICC = 0.98, CI_95_ = 0.95 to 0.99; ICC = 0.92, CI_95_ = 0.78 to 0.97, respectively), and was moderate for number of bucks and headshakes (ICC = 0.65, CI_95_ = 0.22 to 0.86; ICC = 0.73, CI_95_ = 0.4 to 0.9, respectively). Inconsistencies in coding between observers were discussed and resolved prior to coding the full set of videos.

### Statistical analysis

All statistical analysis was performed in R version 4.3.1^37^; [see Supplementary File 6]. Using the power.t.test function (base R), a minimum sample size of 13 calves per treatment was recommended for a power of 0.8, significance level of 0.05, and Cohen’s d at 1.2 for a two-sample t-test. The effect size was determined based on the results from Lecorps et al.^34^ comparing hole-board performance during re-learning by calves that experienced a 50% reduction in milk allowance compared to those who remained at 12 L/d. In the present study, we controlled for within-calf pair variability by matching baited bucket locations. This, along with farm constraints, led our final sample used in the analysis to include 10 calves per treatment.

We used linear mixed-effect models (LMMs, lme4 package v1.1.35.5) to determine whether milk allowance and trial affected calves’ memory scores (working memory, general working memory, and reference memory) and other performance measures (latency to visit the first bucket and trial duration) during both the initial learning (trials 1–14) and re-learning (trials 15–20) phases. We also ran a LMM comparing trials 14 and 15 to determine how memory scores changed from the last trial of initial learning to the first day of re-learning when baited locations were changed. Due to Poisson distribution of vocalisation data, general linear mixed effect models (GLMMs, lme4) were used in place of LMMs. For each measure, one model was built for each phase (initial learning, location change, and re-learning), for a total of 3 models per measure. In the memory score LMMs, we assessed the fit of a random intercept compared to a random slope (trial), as we expected calves to vary in their memory score over the trials (i.e., some calves may improve faster over trials than others). If random slope models returned convergence errors or the AIC did not favour the random slope, random intercepts were used instead. Additionally, when there was an interaction effect (*P* < 0.05) between milk allowance and trial in the memory score models post-hoc analysis was conducted by running one LMM for each treatment with trial as a fixed effect.

We also ran LMMs assessing the effect of milk allowance and trial on time spent play running per minute, and GLMMs (due to Poisson distribution) for point play behaviour with an offset for trial duration. These models were run only for the initial learning and re-learning phases (2 models per measure) as we did not have specific predictions about how play would change alongside baited locations. Ordinal logistic regression (OLR) models were used to assess the effect of milk allowance and trial on the number of baited buckets found (5 levels, 0–4), with one model per phase (3 models total).

In each model, sex (male vs. female), breed (dairy vs. beef), milk allowance (enhanced vs. restricted), trial (as a continuous effect), and an interaction between milk allowance and trial were included as fixed effects. If breed and sex were not significant (*P* > 0.05) they were removed from the model. Interactions were removed from the model if *P* > 0.1. Housing pair and calf ID were included as nested random effects, but in some cases the nested effect caused model singularity, so in these models pair was removed from the random effect. If models still returned singularity with only calf ID as a random effect (usually due to small datasets for baited bucket location change), paired t-tests were run in place of LMMs for each of the enhanced and restricted groups to determine whether the outcome measure differed between trials 14 and 15. For the number of baited buckets found, the interaction was removed if singularity persisted. Degrees of freedom for LMMs were obtained with Satterthwaite’s approximation. Data normality was visualised through histograms; memory scores were normally distributed, and vocalisations and point play were Poisson distributed. All other measures were not normally distributed and were log-transformed (excluding the number of baited buckets found).

## Results

### Initial learning

#### Memory scores

**Fig. 2:**
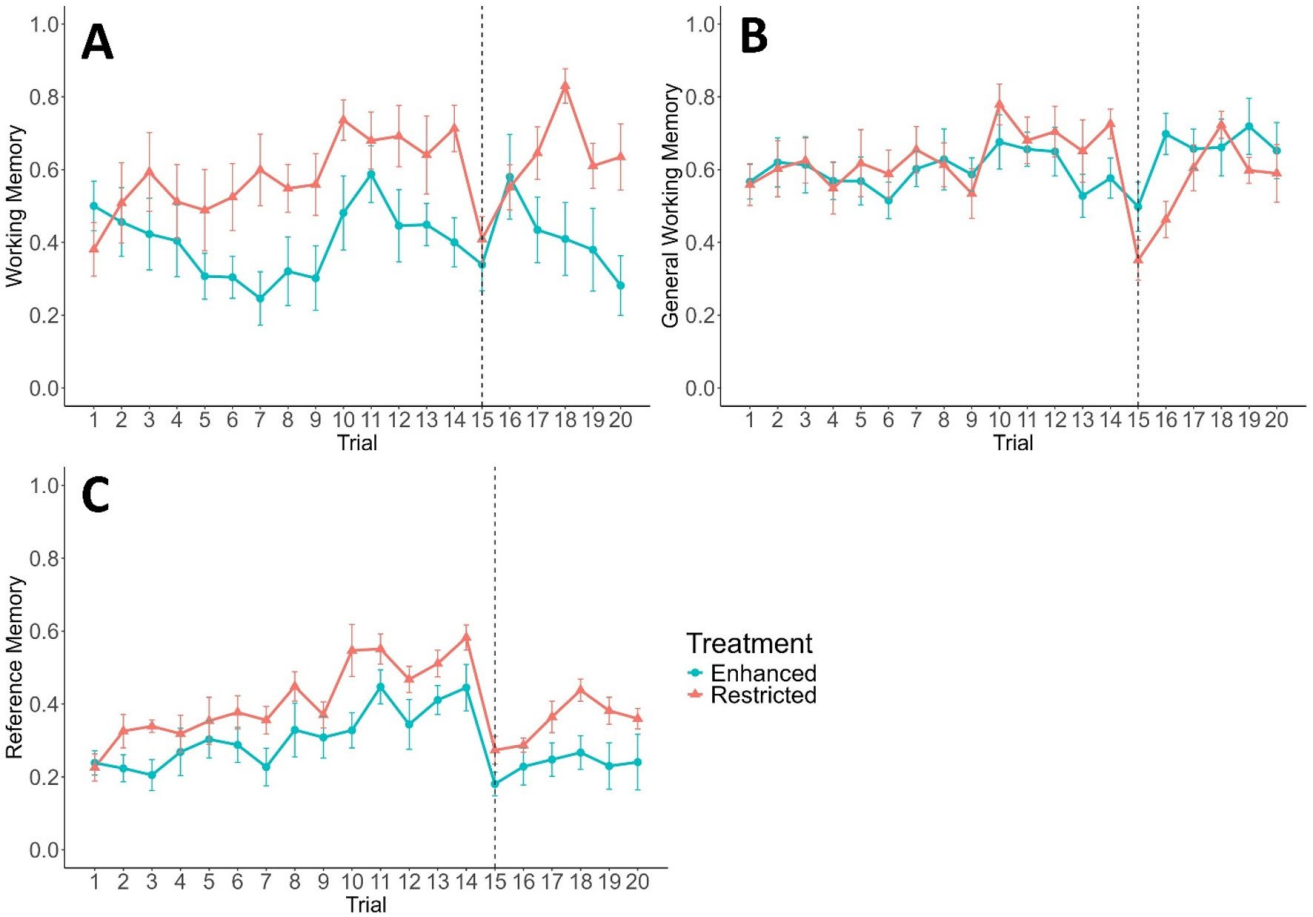
Memory scores calculated from dairy calves’ (*n* = 20) visits during hole-board trials (1–20) for calves fed an enhanced (blue circles) or restricted (orange triangles) milk allowance. Baited bucket locations remained constant from trials 1–14; on trial 15 baited locations were changed (represented by dashed line) and remained constant for the final 6 trials. Working memory and reference memory scores were adjusted to reflect the proportion of baited buckets found. Data are represented as the mean value ± SE. (A) Working memory; (B) General working memory; (C) Reference memory.

During the initial learning phase, calves did not show a decrease in revisits to baited buckets, suggesting their working memory did not improve over time, and working memory was not affected by milk allowance (both *P* > 0.05; Fig. 2A). However, restricted calves’ working memory improved more over time than enhanced calves’ (LMM, t_258_ = 2.39, *P* = 0.018). When separated by milk allowance, restricted calves’ working memory improved over time (LMM, t_129_ = 3.52, *P* < 0.001), but enhanced calves’ did not (*P* > 0.05). Calves made fewer revisits to any bucket within a trial over time, indicating an improvement in general working memory (LMM, t_259_ = 2.2, *P* = 0.029; Fig. 2B), but there was no difference between milk allowances, nor an interaction between the two (both *P* > 0.05). Calves learned the locations of baited buckets over time (reference memory, LMM, t_19_ = 7.93, *P* < 0.001; Fig. 2C) and restricted calves tended to have better reference memory than enhanced calves (t_17.9_ = 1.95, *P* = 0.067), but calves on different milk allowances did not differ in how fast their reference memory improved (*P* > 0.05).

### Other performance measures

**Fig. 3:**
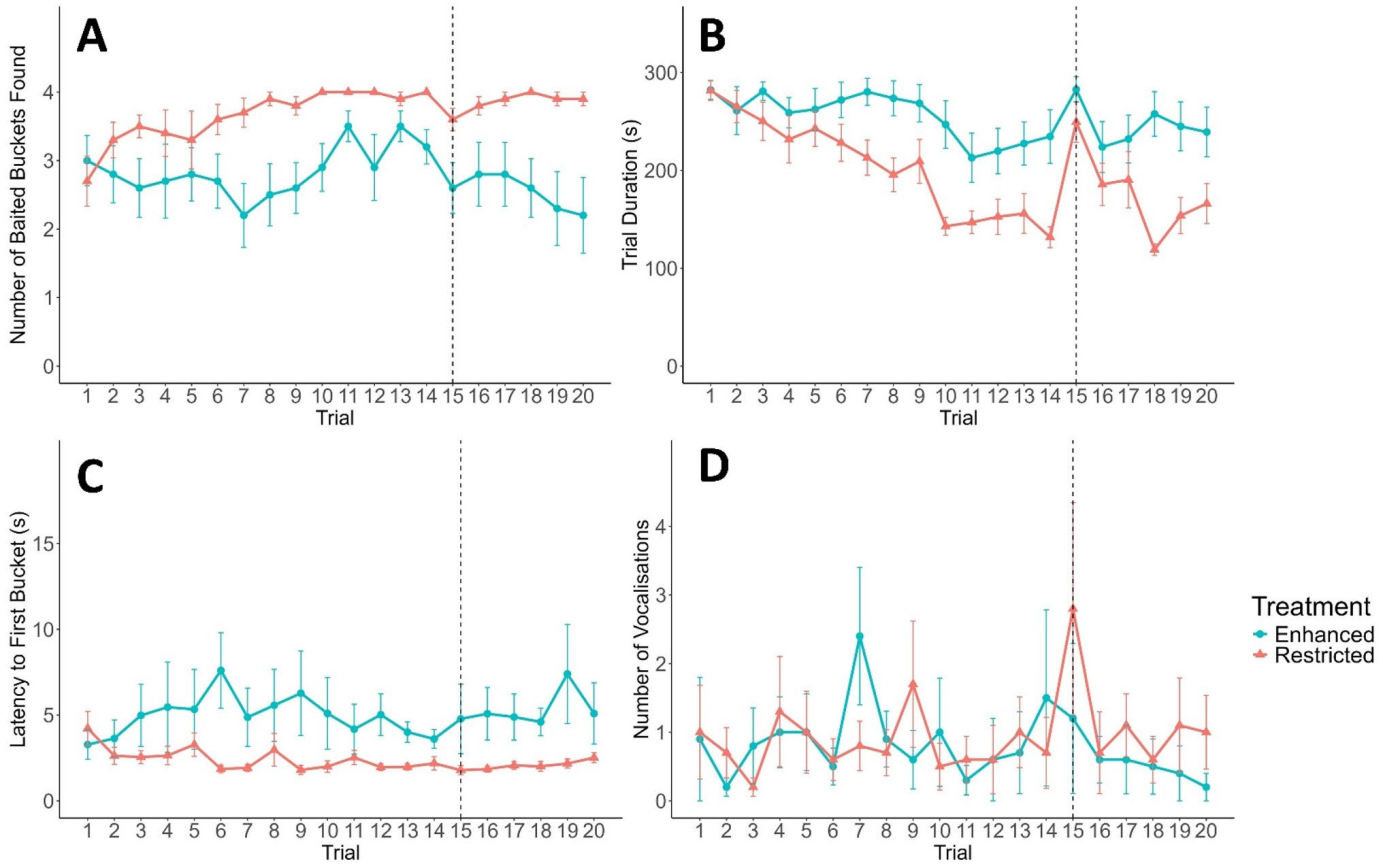
Number of baited buckets found (**A**), trial duration, in seconds (**B**), latency to visit the first bucket, in seconds (**C**), and number of vocalisations (**D**) by hole-board trial (1–20) for dairy calves (*n* = 20) fed an enhanced (blue circles) or restricted (orange triangles) milk allowance. Baited bucket locations remained constant from trials 1–14; on trial 15 baited locations were changed (represented by dashed line) and remained constant for the final 6 trials. Data are represented as the mean value ± SE.

During initial learning, all calves found more baited buckets over time (OLR, t = 2.27, *P* = 0.023; Fig. 3A), but restricted calves found more baited buckets over time compared to enhanced calves (t = 4.06, *P* < 0.001). Milk allowance on its own did not affect the number of baited buckets found (*P* > 0.05). Trial duration was not different between restricted and enhanced calves (*P* > 0.05), but it decreased over time for all calves (LMM, t_255.9_ = -3.84, *P* < 0.001; Fig. 3B). Restricted calves’ trial durations decreased more over time compared to enhanced calves (t_255.5_ = -4.71, *P* < 0.001). Latency to the first bucket was not different between calves on different milk allowances nor across trials (both *P* > 0.05), but restricted calves’ latency decreased more over time (LMM, t_258_ = -2.29, *P* = 0.023; Fig. 3C). There was no effect of milk allowance, trial, or an interaction between the two, on vocalisations during the initial learning phase (both *P* > 0.05; Figure 3D).

### Play behaviour

**Fig. 4:**
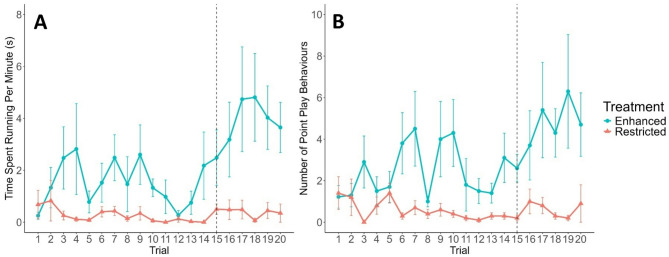
Time spent play running per minute, in seconds (**A**) and number of point play behaviours (**B**) performed by hole-board trial (1–20) for dairy calves (*n* = 20) fed an enhanced (blue circles) or restricted (orange triangles) milk allowance. Baited bucket locations remained constant from trials 1–14; on trial 15 baited locations were changed (represented by dashed line) and remained constant for the final 6 trials. Data are represented as the mean value ± SE.

Restricted calves spent less time running per minute of their trials compared to enhanced calves (LMM, t_17.2_ = -4.64, *P* < 0.001; Fig. 4A), but running did not change over time (*P* > 0.05). Dairy calves ran more than beef calves (t_16.9_ = 3.84, *P* = 0.001). There was no difference between milk allowances in the number of point play behaviours performed on average during initial learning (GLMM, *P* > 0.05; Fig. 4B). In general, all calves performed more point play over time (z = 2.72, *P* = 0.007), but restricted calves played less over time compared to enhanced calves (z = -3.35, *P* < 0.001). Dairy calves performed more point play than beef calves (z = 2.96, *P* = 0.003).

### Response to change in baited locations

#### Memory scores

After changing baited bucket locations, restricted calves made fewer revisits to baited buckets than enhanced calves across both trials, suggesting better working memory (LMM, t_18.1_ = 2.16, *P* = 0.044; Fig. 2A), but their working memory tended to decrease more than that of enhanced calves (t_18_ = -2.06, *P* = 0.055). On average for all calves, working memory was not different across trials (*P* > 0.05). Restricted calves made fewer revisits to all buckets than enhanced calves (general working memory, LMM, t_18.1_ = 2.85, *P* = 0.011; Fig. 2B). From trial 14 to 15 restricted calves’ general working memory decreased more compared to enhanced calves (t_18_ = -2.85, *P* = 0.011). Trial did not affect general working memory (*P* > 0.05). When separated by milk allowance, restricted calves revisited more buckets after baited location change (t_18_ = -5.47, *P* < 0.001), but enhanced calves did not (*P* > 0.05). After the baited locations changed, ability to discriminate between baited and unbaited locations (reference memory) decreased among both restricted (t-test, t_9_ = 5.37, *P* < 0.001; Fig. 2C) and enhanced (t-test, t_9_ = 3.42, *P* = 0.0038) calves.

### Other performance measures

When baited bucket locations were changed, the number of baited buckets found decreased for all calves (OLR, t = -2.28, *P* = 0.023; Fig. 3A), but over both trials restricted calves found more baited buckets (t = 2.67, *P* = 0.0077). Trial and milk allowance did not interact (*P* > 0.05). Restricted calves had shorter trial durations than enhanced calves when the baited locations changed (LMM, t_18.1_ = -2.15, *P* = 0.045; Fig. 3B), and trial duration tended to increase among all calves between the two trials (t_18_ = 1.92, *P* = 0.071). Restricted calves’ trial duration tended to increase more when baited locations changed compared to enhanced calves (t_18_ = 2.03, *P* = 0.058). Latency to the first bucket did not differ after baited locations changed (t-tests, *P* > 0.05 for both treatments; Fig. 3C). Restricted calves vocalised less than enhanced calves on average across both trials (GLMM, z = -2.61, *P* = 0.0092; Fig. 3D) but restricted calves vocalised more when baited locations changed (z = 2.81, *P* = 0.005), and trial had no effect (*P* > 0.05).

### Re-learning phase

#### Memory scores

During the re-learning phase, calves’ revisits to baited buckets (working memory) did not change over time (*P* > 0.05; Fig. 2A). Restricted calves had better working memory than enhanced calves (LMM, t_100.9_ = -2.32, *P* = 0.022), but restricted calves’ scores improved more over time (t_97.9_ = 2.83, *P* = 0.006). When separated by milk allowance, over time restricted calves’ working memory improved (LMM, t_49_ = 2.61, *P* = 0.012), but there was no indication of this improvement in enhanced calves (*P* > 0.05). Calves in both treatments made fewer revisits to buckets over time, indicating an improvement in general working memory (LMM, t_98.9_ = 3.82, *P* < 0.001; Fig. 2B). There was no difference between milk allowances and no interaction between trial and milk allowance (both *P* > 0.05). Restricted calves were better than enhanced calves at remembering the baited bucket locations, evidenced by higher reference memory scores (LMM, t_18_ = 2.5, *P* = 0.022; Fig. 2C), and reference memory improved over time for all calves (t_99_ = 2.78, *P* = 0.007).

### Other performance measures

During the re-learning phase, the number of baited buckets found did not differ between milk allowances or trials (both *P* > 0.05; Fig. 3A) but restricted calves found more baited buckets over time than enhanced calves (OLR, t = 2.16, *P* = 0.031). Milk allowance and trial on their own had no effect on trial duration (both *P* > 0.05; Fig. 3B), but restricted calves completed trials faster over time compared to enhanced calves (LMM, t_98_ = -2.23, *P* = 0.028). Restricted calves were faster to visit the first bucket than enhanced calves (LMM, t_18_ = -2.94, *P* = 0.009; Fig. 3C), but latency increased over time for all calves (t_99_ = 2.97, *P* = 0.004). On average, calves vocalised less over time (GLMM, z = -3.88, *P* < 0.001; Fig. 3D), restricted calves tended to vocalise more (z = 1.72, *P* = 0.085), and there was no interaction between trial and milk allowance (*P* > 0.05).

### Play behaviour

During the re-learning phase, time spent running per minute increased over time for all calves (LMM, t_98_ = 2.12 *P* = 0.036; Fig. 4A), and restricted calves ran less over time compared to enhanced calves (t_98_ = -2.01, *P* = 0.047). On average, enhanced and restricted calves did not differ in time spent running during re-learning (*P* > 0.05). All calves performed more point play over time (GLMM, z = 2.96, *P* = 0.003), but restricted calves played less than enhanced calves (z = -3.83, *P* < 0.001).

## Discussion

Calves who were feed-restricted generally had better working memory (within-trial memory of which baited buckets they had drank from) and reference memory (memory across trials of the baited bucket locations) compared to enhanced-fed calves, but there was no difference in general working memory (within-trial memory of which buckets they had already visited) between milk allowances. Enhanced calves performed more of both play types than restricted calves.

Contrary to our expectations, and the findings from other research that feed restriction negatively impacts farm animal cognition (e.g.^26,28^, feed-restricted calves did not demonstrate reduced cognitive performance in a hole-board task compared to calves who were fed more milk. Restricted calves demonstrated initial learning and re-learning patterns similar to other studies assessing hole-board performance in dairy calves, where the control animals received 8–12 L/day^28,34,35^. In contrast, enhanced calves demonstrated slower increases in memory scores and typically did not recover as quickly after the baited location change, and in the case of working memory their scores declined. Studies looking at the impact of affective manipulations on cognitive performance in the hole-board test have yielded contrasting results. Yoo et al.^35^ did not find cognitive impairments in a hole-board task among dairy calves who were disbudded compared to sham-disbudded ones and those who received additional pain mitigation, despite research demonstrating that long lasting pain affects memory in humans^38^ and rats^39^. In pigs, environmental enrichment improved performance in a hole-board task^40^, while introduction to an unfamiliar pig impaired spatial memory^41^, but mixing stress had no effect^42^. In two different studies assessing hole-board performance between aviary- and cage-raised hens, one study reported better working memory during reversal learning in aviary hens^43^, whereas the other found higher reference memory among aviary hens only in the initial learning phase^44^. While Conrad^19^ reviewed the effects of social stress on hole-board performance in male rats, a similar review in farm animals covering the range of stressors they face due to routine husbandry has not been conducted. Therefore, it is sometimes unclear how affective states or treatments expected to have emotional consequences may impact the animal’s performance in this test.

Our results may be better explained by differences in food motivation rather than cognitive processes, with restricted calves being more motivated to find the baited locations compared to the enhanced group. Success in a spatial foraging task requires the animal to be motivated to find food rewards^18^, and it is expected that feed restriction increases food motivation with evidence in dairy cows^45,46^ and calves^6^. Food motivation can drive hungry animals to be more successful in food reward-based learning tasks compared to less motivated counterparts; for example, pheasant chicks’ participation and success in an operant foraging task is predicted by motivation to engage in the test and find reward worms^47^, and quail imitate demonstrator behaviour (peck or step a treadle for a food reward) only when they are hungry at the time of observing the demonstrator bird^48^. In this study, restricted calves had shorter trial durations and latencies to the first bucket (or their trial duration and latencies decreased more over time), and they found more of the baited buckets as trials progressed, indicating that they were highly motivated to find food and participate in the test compared to enhanced calves. We expected that restricted calves may have been too hungry to perform well in the hole-board test due to the severity of feed restriction combined with 16 h of food deprivation at the time of testing, and that this length of food deprivation would be sufficient to ensure enhanced calves were hungry enough to be food-motivated at the time of the test. However, the significantly higher play motivation and lower food motivation among enhanced calves limits our ability to infer the affective state experienced by restricted calves. Their cognitive performance could not be meaningfully compared to enhanced calves, who were much less motivated to engage in the task.

While enhanced-fed calves were not as motivated to engage in the hole-board test, they were highly motivated to play, and played more than restricted calves during all phases of the test. Play running in calves is expected to increase with higher milk intake^33^, and calves play more in a novel arena when they are housed in spatially restrictive environments compared to calves given more space^30^. The hole-board arena was nearly triple the size of calves’ outdoor access in their home-pens, giving them an opportunity to express play behaviours not allowed in their home-pen where they were spatially restricted (although housed as per the requirements of the Welfare of Farmed Animals (England) Regulations 2007). Clearly, play was highly important to enhanced-fed calves, because despite drinking most or all of their morning meal (6 L) immediately after the hole-board test (indicating they were hungry and motivated to drink), they did not prioritise finding milk over playing during the test, especially compared to feed-restricted calves.

Our results demonstrate that calves’ priorities shifted from foraging to play when they were not feed-restricted. Hunger is expected to impact trade-offs that animals make between food and other rewards. Verbeek et al.^49^ reported that feed-restricted ewes were more optimistic than higher-fed ewes in a judgement bias task, but the authors concluded that optimistic behaviour in hungry ewes was likely due to increased motivation to find food rather than more positive affective state. Additionally, research in mice has shown that hunger suppresses other competing motivations, including thirst, anxiety and fear responses, and social interactions when food is accessible^50^. While less research has been conducted on trade-offs between foraging and play, Muller-Schwarze et al.^51^ found that feed restriction decreased play behaviour and increased grazing behaviour in deer fawns, providing some evidence that young animals may prioritise foraging over play opportunities when they are hungry. Although we cannot conclude whether feed-restricted calves show cognitive deficits, the suppression of play among these animals may indicate reduced welfare. Engaging in play helps young mammals build emotional resilience to stressful or unexpected situations^52,53^. Suppressed play is often linked to poor welfare or negative affect^54,55^. Indeed, play is reduced in calves when they are subjected to painful procedures^56,57^ and feed restriction^32^. The fact that the latter prioritised play over foraging despite 16 h of feed deprivation (following the normal feeding schedule on the dairy farm, but calves normally feed over 7 meals interspersed throughout the day when fed *ad libitum*;^3^ highlights the importance of play to young calves.

The findings of this study provide insight into how feed restriction impacts calves’ motivation to engage in a spatial foraging task versus play opportunities, which opens an avenue for future research to explore conflicting motivations and how animals prioritise them. Research on how hunger interacts with other motivations has been largely conducted in laboratory rodents, under heavily controlled conditions, and motivational systems are often studied in isolation or pairs^58^. There is a need for this research to be conducted under more naturalistic conditions where animals are confronted with multiple drives simultaneously^58^. This gap widens further in farm animals, where little research has been conducted on how these animals, who are often subjected to management that limits their ability to express certain behaviours, deal with conflicting motivations. Given that feed restriction is common in many farm systems^9^, we recommend that future research examine how hunger may suppress other motivations (e.g., social behaviour) under different husbandry conditions. Future research could reduce the interference of play motivation during testing by allowing calves to satisfy their play motivation outside of the hole-board test, such as by using less spatially restrictive housing or providing opportunities to play in an arena.

## Conclusions

This study investigated whether feed-restricted dairy calves exhibit deficits in cognitive performance in a hole-board task. In contrast with our expectations, feed-restricted calves generally exhibited better test performance than enhanced-fed calves, but these differences appear to have been driven by different prioritisation of behaviour rather than differences in cognitive function. Enhanced-fed calves engaged in more play than feed-restricted ones, indicating that calves prioritised play over finding food when feed restrictions were absent, whereas hunger suppressed feed-restricted calves’ motivation to engage in play and foraging took precedence instead. This study highlights how hunger can suppress competing motivations in young calves, and opens an avenue to explore how animals prioritise motivations when they conflict.

## Supplementary Information

Below is the link to the electronic supplementary material.

Supplementary Material 1

## Data Availability

Data is provided within the manuscript or supplementary information files.

## References

[1] Mahendran, S., Wathes, D., Booth, R. & Blackie, N. A survey of calf management practices and farmer perceptions of calf housing in UK dairy herds. *J. Dairy Sci.* **105**, 409–423. 10.3168/jds.2021-20638 (2022). 34763915 10.3168/jds.2021-20638

[2] Appleby, M. C., Weary, D. M. & Chua, B. Performance and feeding behaviour of calves on ad libitum milk from artificial teats. *Appl. Anim. Behav. Sci.* **74** (3), 191–201. 10.1016/S0168-1591(01)00171-X (2001).

[3] Miller-Cushon, E. K., Bergeron, R., Leslie, K. E. & DeVries, T. J. Effect of milk feeding level on development of feeding behavior in dairy calves. *J. Dairy Sci.* **96** (1), 551–564. 10.3168/JDS.2012-5937 (2013). 23164223 10.3168/jds.2012-5937

[4] Welk, A., Otten, N. D. & Jensen, M. B. Invited review: The effect of milk feeding practices on dairy calf behavior, health, and performance—A systematic review. *J. Dairy Sci.* **106** (9), 5853–5879. 10.3168/JDS.2022-22900 (2023). 37474370 10.3168/jds.2022-22900

[5] Khan, M. A., Weary, D. M. & Von Keyserlingk, M. A. G. Invited review: Effects of milk ration on solid feed intake, weaning, and performance in dairy heifers. *J. Dairy Sci.* **94** (3), 1071–1081. 10.3168/JDS.2010-3733 (2011). 21338773 10.3168/jds.2010-3733

[6] De Paula Vieira, A., Guesdon, V., de Passille, A. M., von Keyserlingk, M. A. G. & Weary, D. M. Behavioural indicators of hunger in dairy calves. *Appl. Anim. Behav. Sci.* **109**, 180–189. 10.1016/j.applanim.2007.03.006 (2008).

[7] Ackermans, M. A., Jonker, N. C., Bennik, E. C. & de Jong, P. J. Hunger increases negative and decreases positive emotions in women with a healthy weight. *Appetite* **168**, 105746. 10.1016/J.APPET.2021.105746 (2022). 34637770 10.1016/j.appet.2021.105746

[8] hangry *Oxford English Dictionary*. (2023). 10.1093/OED/1081659877

[9] D’Eath, R. B., Tolkamp, B. J., Kyriazakis, I. & Lawrence, A. B. Freedom from hunger’ and preventing obesity: the animal welfare implications of reducing food quantity or quality. *Anim. Behav.* **77** (2), 275–288. 10.1016/J.ANBEHAV.2008.10.028 (2009).

[10] Duncan, I. J. H. A Concept of Welfare Based on Feelings. *Well-Being Farm. Animals: Challenges Solutions*. 85–101. 10.1002/9780470344859.CH5 (2004).

[11] Paul, E. S., Harding, E. J. & Mendl, M. Measuring emotional processes in animals: the utility of a cognitive approach. *Neurosci. Biobehavioral Reviews*. **29** (3), 469–491. 10.1016/J.NEUBIOREV.2005.01.002 (2005). 10.1016/j.neubiorev.2005.01.002 15820551

[12] Pizzagalli, D. A., Iosifescu, D., Hallett, L. A., Ratner, K. G. & Fava, M. Reduced hedonic capacity in major depressive disorder: Evidence from a probabilistic reward task. *J. Psychiatr. Res.* **43** (1), 76–87. 10.1016/J.JPSYCHIRES.2008.03.001 (2008). 18433774 10.1016/j.jpsychires.2008.03.001 PMC2637997

[13] Slaney, C. L., Hales, C. A. & Robinson, E. S. J. Rat models of reward deficits in psychiatric disorders. *Curr. Opin. Behav. Sci.* **22**, 136–142. 10.1016/J.COBEHA.2018.05.001 (2018). 30123817 10.1016/j.cobeha.2018.05.001 PMC6095230

[14] Baciadonna, L. & McElligott, A. G. The use of judgement bias to assess welfare in farm livestock. *Anim Welf.* **24** (1), 81–91. 10.7120/09627286.24.1.081 (2015).

[15] Lagisz, M. et al. Optimism, pessimism and judgement bias in animals: A systematic review and meta-analysis. *Neurosci. Biobehavioral Reviews*. **118**, 3–17. 10.1016/J.NEUBIOREV.2020.07.012 (2020). 10.1016/j.neubiorev.2020.07.012 32682742

[16] Neville, V. et al. Pharmacological manipulations of judgement bias: A systematic review and meta-analysis. *Neurosci. Biobehavioral Reviews*. **108**, 269–286. 10.1016/J.NEUBIOREV.2019.11.008 (2020). 10.1016/j.neubiorev.2019.11.008 PMC6966323 31747552

[17] Hinchcliffe, J. K. & Robinson, E. S. J. The Affective Bias Test and Reward Learning Assay: Neuropsychological Models for Depression Research and Investigating Antidepressant Treatments in Rodents. *Curr. Protocols*. **4** (6), e1057. 10.1002/CPZ1.1057 (2024). 10.1002/cpz1.1057 38923877

[18] van der Staay, F. J., Gieling, E. T., Pinzón, N. E., Nordquist, R. E. & Ohl, F. The appetitively motivated cognitive holeboard: A family of complex spatial discrimination tasks for assessing learning and memory. *Neurosci. Biobehavioral Reviews*. **36** (1), 379–403. 10.1016/J.NEUBIOREV.2011.07.008 (2012). 10.1016/j.neubiorev.2011.07.008 21810442

[19] Conrad, C. D. A critical review of chronic stress effects on spatial learning and memory. *Prog. Neuropsychopharmacol. Biol. Psychiatry*. **34** (5), 742–755. 10.1016/J.PNPBP.2009.11.003 (2010). 19903505 10.1016/j.pnpbp.2009.11.003

[20] Mendl, M., Burman, O., Laughlin, K. & Paul, E. Animal Memory and Animal Welfare. *Anim Welf.* **10** (S1), S141–S159. 10.1017/S0962728600023587 (2001).

[21] Day, J. E. L., Kyriazakis, I. & Lawrence, A. B. The effect of food deprivation on the expression of foraging and exploratory behaviour in the growing pig. *Appl. Anim. Behav. Sci.* **42** (3), 193–206. 10.1016/0168-1591(95)93889-9 (1995).

[22] Smith, N. K. & Grueter, B. A. Hunger-driven adaptive prioritization of behavior. *FEBS J.* **289** (4), 922–936. 10.1111/FEBS.15791 (2022). 33630426 10.1111/febs.15791

[23] Krashes, M. J. et al. Rapid, reversible activation of AgRP neurons drives feeding behavior in mice. *J. Clin. Invest.* **121** (4), 1424–1428. 10.1172/JCI46229 (2011). 21364278 10.1172/JCI46229 PMC3069789

[24] Lawrence, A. B., Appleby, M. C. & Macleod, H. A. Measuring hunger in the pig using operant conditioning: The effect of food restriction. *Anim. Sci.* **47** (1), 131–137. 10.1017/S0003356100037132 (1988).

[25] Mendl, M. Performing under pressure: stress and cognitive function. *Appl. Anim. Behav. Sci.* **65** (3), 221–244. 10.1016/S0168-1591(99)00088-X (1999).

[26] Buckley, L. A. et al. Too hungry to learn? Hungry broiler breeders fail to learn a Y-maze food quantity discrimination task. *Anim Welf.* **20** (4), 469–481. 10.1017/S0962728600003110 (2011).

[27] Fu, Y. et al. Food restriction affects Y-maze spatial recognition memory in developing mice. *Int. J. Dev. Neurosci.* **60**, 8–15. 10.1016/j.ijdevneu.2017.03.010 (2017). 28377130 10.1016/j.ijdevneu.2017.03.010

[28] Lecorps, B., Woodroffe, R. E., von Keyserlingk, M. A. G. & Weary, D. M. Hunger affects cognitive performance of dairy calves. *Biology Letter*. **19**, 20220475. 10.1098/rsbl.2022.0475 (2023). 10.1098/rsbl.2022.0475 PMC9845969 36651027

[29] Jensen, M. B. & Kyhn, R. Play behaviour in group-housed dairy calves, the effect of space allowance. *Appl. Anim. Behav. Sci.* **67** (1–2), 35–46. 10.1016/S0168-1591(99)00113-6 (2000). 10719187 10.1016/s0168-1591(99)00113-6

[30] Rushen, J. & de Passille, A. M. Locomotor play of veal calves in an arena: Are effects of feed level and spatial restriction mediated by responses to novelty? *Appl. Anim. Behav. Sci.* **155**, 34–41. https://doi-org.bris.idm.oclc.org/10.1016/j.applanim.2014.03.009

[31] Sutherland, M. A., Worth, G. M., Schütz, K. E. & Stewart, M. Rearing substrate and space allowance influences locomotor play behaviour of dairy calves in an arena test. *Appl. Anim. Behav. Sci.* **154**, 8–14. 10.1016/J.APPLANIM.2014.02.008 (2014).

[32] Jensen, M. B., Duve, L. R. & Weary, D. M. Pair housing and enhanced milk allowance increase play behavior and improve performance in dairy calves. *J. Dairy Sci.* **98** (4), 2568–2575. 10.3168/JDS.2014-8272 (2015). 25682142 10.3168/jds.2014-8272

[33] Krachun, C., Rushen, J. & Marie De Passillé, A. Play behaviour in dairy calves is reduced by weaning and by a low energy intake. *Appl. Anim. Behav. Sci.* **122**, 71–76. 10.1016/j.applanim.2009.12.002 (2010).

[34] Lecorps, B., Woodroffe, R. E., von Keyserlingk, M. A. G. & Weary, D. M. Assessing cognitive performance in dairy calves using a modified hole-board test. *Anim. Cogn.* **25** (5), 1365–1370. 10.1007/s10071-022-01617-5 (2022). 35347498 10.1007/s10071-022-01617-5

[35] Yoo, S., von Keyserlingk, M. A. G. & Weary, D. M. The effects of pain following disbudding on calf memory. *J. Dairy Sci.* **106**, 9507–9513. 10.3168/jds.2023-23604 (2023). 37678789 10.3168/jds.2023-23604

[36] Friard, O. & Gamba, M. BORIS: a free, versatile open-source event-logging software for video/audio coding and live observations. *Methods Ecol. Evol.* **7**, 1325–1330 (2016).

[37] Posit team. *RStudio: Integrated Development Environment for R. Posit Software* (PBC, 2024). http://www.posit.co/

[38] Mazza, S., Frot, M. & Rey, A. E. A comprehensive literature review of chronic pain and memory. *Progress Neuropsychopharmacol. Biol. Psychiatry*. **87**, 183–192. 10.1016/j.pnpbp.2017.08.006 (2018). https://doi.org/https://doi-org.bris.idm.oclc. 10.1016/j.pnpbp.2017.08.006 28797640

[39] Johnston, I. N., Maier, S. F., Rudy, J. W. & Watkins, L. R. Post-conditioning experience with acute or chronic inflammatory pain reduces contextual fear conditioning in the rat. *Behav. Brain. Res.* **226** (2), 361–368. 10.1016/J.BBR.2011.08.048 (2012). 21920390 10.1016/j.bbr.2011.08.048 PMC5652308

[40] Grimberg-Henrici, C. G. E., Vermaak, P., Bolhuis, J. E., Nordquist, R. E. & Van Der Staay, F. J. Effects of environmental enrichment on cognitive performance of pigs in a spatial holeboard discrimination task. *Anim. Cogn.* **19**, 271–283. 10.1007/s10071-015-0932-7 (2016). 26520648 10.1007/s10071-015-0932-7 PMC4751158

[41] Laughlin, K., Huck, M. & Mendl, M. Disturbance effects of environmental stimuli on pig spatial memory. *Appl. Anim. Behav. Sci.* **64** (3), 169–180. 10.1016/S0168-1591(99)00036-2 (1999).

[42] Arts, J. W. M., van der Staay, F. J. & Ekkel, E. D. Working and reference memory of pigs in the spatial holeboard discrimination task. *Behav. Brain. Res.* **205** (1), 303–306. 10.1016/J.BBR.2009.06.014 (2009). 19539660 10.1016/j.bbr.2009.06.014

[43] Tahamtani, F. M., Nordgreen, J., Nordquist, R. E. & Janczak, A. M. Early life in a barren environment adversely affects spatial cognition in laying hens (Gallus gallus domesticus). *Front. Veterinary Sci.* **2** (3). 10.3389/FVETS.2015.00003/BIBTEX (2015). 10.3389/fvets.2015.00003 PMC4672168 26664932

[44] Dumontier, L., Janczak, A. M., Smulders, T. V. & Nordgreen, J. Effects of the rearing environment complexity on laying hens’ spatial cognition: A holeboard test approach. *Appl. Anim. Behav. Sci.* **260**, 105878. 10.1016/J.APPLANIM.2023.105878 (2023).

[45] Franchi, G. A., Herskin, M. S. & Jensen, M. B. Dairy cows fed a low energy diet before dry-off show signs of hunger despite ad libitum access. *Sci. Rep.* **9** (1), 1–9. 10.1038/s41598-019-51866-7 (2019). 31695053 10.1038/s41598-019-51866-7 PMC6834606

[46] Schütz, K., Davison, D. & Matthews, L. Do different levels of moderate feed deprivation in dairy cows affect feeding motivation? *Appl. Anim. Behav. Sci.* **101** (3–4), 253–263. 10.1016/J.APPLANIM.2006.02.008 (2006).

[47] van Horik, J. O. & Madden, J. R. A problem with problem solving: motivational traits, but not cognition, predict success on novel operant foraging tasks. *Anim. Behav.* **114**, 189–198. 10.1016/J.ANBEHAV.2016.02.006 (2016). 27122637 10.1016/j.anbehav.2016.02.006 PMC4833691

[48] Dorrance, B. R. & Zentall, T. R. Imitative learning in Japanese quail (Coturnix japonica) depends on the motivational state of the observer quail at the time of observation. *J. Comp. Psychol.* **115** (1), 62–67. 10.1037/0735-7036.115.1.62 (2001). 11334220 10.1037/0735-7036.115.1.62

[49] Verbeek, E., Ferguson, D. & Lee, C. Are hungry sheep more pessimistic? The effects of food restriction on cognitive bias and the involvement of ghrelin in its regulation. *Physiol. Behav.* **123**, 67–75. 10.1016/J.PHYSBEH.2013.09.017 (2014). 24096007 10.1016/j.physbeh.2013.09.017

[50] Burnett, C. J. et al. Hunger-Driven Motivational State Competition. *Neuron* 92(1), 187–201. (2016). 10.1016/J.NEURON.2016.08.032/ATTACHMENT/BC41658A-7E5D-4258-8C5D-C202D16A5E5B/MMC2.PDF 10.1016/j.neuron.2016.08.032 PMC5082717 27693254

[51] Muller-Schwarze, D., Stagge, B. & Muller-Schwarze, C. Play behavior: Persistence, decrease, and energetic compensation during food shortage in deer fawns. *Science* **215** (4528), 85–87. 10.1126/SCIENCE.215.4528.85 (1982). 17790473 10.1126/science.215.4528.85

[52] Špinka, M., Newberry, R. C. & Bekoff, M. Mammalian play: Training for the unexpected. *Q. Rev. Biology*. **76** (2), 141–168. 10.1086/393866;WGROUP:STRING:PUBLICATION (2001). 10.1086/393866 11409050

[53] Wallis, A., Mendl, M., Lecorps, B. & Held, S. From welfare indicator to welfare contributor: the role of play in building flexibility and resilience in captive animals. *Proceedings B* 292(2059), 20251962. (2025). 10.1098/rspb.2025.1962 10.1098/rspb.2025.1962 PMC12651949 41295905

[54] Ahloy-Dallaire, J., Espinosa, J. & Mason, G. Play and optimal welfare: Does play indicate the presence of positive affective states? *Behavioural Processes* 156, 3–15. (2018). 10.1016/J.BEPROC.2017.11.011 10.1016/j.beproc.2017.11.011 29155308

[55] Held, S. D. E. & Špinka, M. Animal play and animal welfare. *Anim. Behav.* **81** (5), 891–899. 10.1016/J.ANBEHAV.2011.01.007 (2011).

[56] Mintline, E. M. et al. Play behavior as an indicator of animal welfare: Disbudding in dairy calves. *Appl. Anim. Behav. Sci.* **144** (1–2), 22–30. 10.1016/J.APPLANIM.2012.12.008 (2013).

[57] Winder, C. B. et al. Clinical trial of local anesthetic protocols for acute pain associated with caustic paste disbudding in dairy calves. *J. Dairy Sci.* **100** (8), 6429–6441. 10.3168/JDS.2017-12724 (2017). 28551190 10.3168/jds.2017-12724

[58] Sutton Hickey, A. K. & Krashes, M. J. Integrating Hunger with Rival Motivations. *Trends Endocrinol. Metabolism*. **31** (7), 495–507. 10.1016/J.TEM.2020.04.006 (2020). 10.1016/j.tem.2020.04.006 32387196

