# Supplementary material for: Calves fed more milk prioritise play over feeding in a hole-board test
Source: Sci Rep. 2026 Apr 13;16:17243. doi: 10.1038/s41598-026-47403-y (PMC13234365; doi:10.1038/s41598-026-47403-y)
Supplement: Supplementary file 1 — Supplementary Material 1 [file 41598_2026_47403_MOESM1_ESM.zip › Supplementary materials/Holeboard_Ethogram.docx]

| **Behaviour** | **Description** | **Behaviour type** |
| --- | --- | --- |
| Play run | Calf exhibits fast and sustained forward motion with at least two hooves lifted from the ground simultaneously (e.g., galloping, cantering, trotting). At least two distinct movement patterns must occur. 3 seconds must pass between the end of the movement and the next visit to a bucket. | Duration |
| Jump | Calf’s front legs lift from the ground with the calf moving in an upward and/or forward motion. Hind legs lift from the ground towards the end of the movement. | Point |
| Buck | Calf kicks up both hind legs to the back or side of the body. | Point |
| Head shake | Calf rapidly moves head from side to side or rotates it. | Point |

*Table 1. Ethogram for measuring play behaviour during hole-board testing trials. Behaviours adapted from Jensen and Khyn (2000).*
